# Effects of Levistilide A on Hemorheology and Endothelial Cell Injury in Rats with Blood Stasis

**DOI:** 10.1155/2021/6595383

**Published:** 2021-12-02

**Authors:** XiaoTong Liu, MiJia Zhang, YuJiao Li, WenLu He, GuangHua Lu, Qiong Wang, QiaoZhi Wang

**Affiliations:** ^1^Department of Histology and Embryology, School of Basic Medical Sciences, Southwest Medical University (SWMU), Luzhou 646000, Sichuan, China; ^2^Sino-Portugal TCM International Cooperation Center, The Affiliated Traditional Chinese Medicine Hospital of Southwest Medical University, Luzhou 646000, Sichuan, China; ^3^School of Ethnic Medicine, Chengdu University of Traditional Chinese Medicine, Chengdu 611137, Sichuan, China; ^4^Institute of Food Science and Technology, Chinese Academy of Agricultural Sciences (CAAS), Beijing 100193, China

## Abstract

**Background:**

Vascular endothelial cell injury is not only the initiating factor of cardiovascular and cerebrovascular diseases but also the essence of blood stasis. Levistilide A (LA), a natural component isolated from the traditional Chinese herb, *Ligusticum chuanxiong* Hort, has traditional effects on improving blood circulation and removing stasis. In this study, the effects and potential mechanisms of LA in the rat model of blood stasis and the mechanism in endothelial cell injury have been explored.

**Materials and Methods:**

In this experiment, the effects of LA on the model of acute blood stasis in rats were explored. The blood samples were collected for the measurement of coagulation and hemorheological indices, and the carotid arteries were also excised from rats for hematoxylin-eosin (HE) staining and immunohistochemistry (IHC). In addition, the improvement effects of LA on the H_2_O_2_-induced human umbilical vein endothelial cell (HUVEC) injury model were evaluated. And the cell viability detection was conducted by the CCK8 assay, and the pathway-related protein expressions were detected by western blotting.

**Results:**

*In vivo*, compared with the model group, the treatment of LA (10 mg/kg) could reduce the FIB (fibrinogen) content (*P* < 0.01), increase the INR (international normalized ratio) and PT (prothrombin time) (*P* < 0.01), and reduce the plasma viscosity (*P* < 0.05) and whole blood viscosities of low, medium, and high shear rates in the blood of blood stasis model rats (*P* < 0.01). *In vitro*, the cell viability in the LA-pretreated group was higher than that of the model group (*P* < 0.05). The expression levels of PI3K, AKT, and eNOs in the LA-pretreated group were increased (*P* < 0.01) as compared to the model group.

**Conclusion:**

These findings demonstrated that LA has the ability to improve blood hypercoagulation and blood viscosity, and enhance the viability of cells. It is more likely that it exerts a protective effect on the endothelial cell through the PI3K-AKT-eNOs pathway. These results indicate LA will be a potential candidate to cure blood stasis with endothelial cell injury.

## 1. Introduction

Blood stasis is a syndrome caused by the blood stagnation in the vessels or the blood overflowing outside the vessel caused by various factors that is stored in the body and failed to be discharged [1]. The modern medicine mainly observes abnormal blood indicators, which are related to blood stasis including blood thickness, stickiness and coagulation, aggregation, morphological changes, and abnormal functioning. The research group of Academician at Chen Keji used a combination of traditional Chinese medicine (TCM) and modern medicine, for the analysis of blood stasis, expounding its scientific connotation and the mechanism of action of TCM for improvement in the blood circulation and elimination of blood stasis. These are reflected as the improvement of physical and chemical properties of blood, functioning of blood vessels, attenuation of blood thickness, and related phenomenon (anticoagulation, inhibition of platelet aggregation, antithrombosis, etc.) and developed the theory of blood stasis. This added a new dimension in the modern medical research to enhance blood circulation and removal of blood stasis [2].

The integrity of vascular endothelial cells lining the inner surface of the cardiovascular system is crucial for maintaining normal physiological functions. Vascular endothelial cells perform multiple functions such as maintenance of normal blood circulation and hemostasis between multiple active substances [3, 4]. For example, coagulation balance is maintained by antithrombin (AT), thrombomodulin (TM), tissue factor pathway inhibitor (TFPI), nitric oxide (NO), endothelin (ET), etc., emphasizing the nature complexities of activities governed by endothelial cells that serve as the main site for their synthesis and release [5, 6]. Blood stasis often occurs with a variety of syndromes, leading to hemorheological changes and microcirculation disorders, as well as inflammation and disruption in the immune response. Consequently, cytokines and inflammatory mediators lead to endothelial damage disrupting endothelial normal functioning. Hence, an early sign of changing vascular homeostasis can be used as a predictor of cardiovascular events related to the development of cardiovascular and cerebrovascular diseases [7, 8]. The Consensus on the Diagnosis and Treatment of Blood Stasis with Integrated Traditional Chinese and Western Medicine proposes that blood rheology and coagulation function, NO and ET levels, platelet aggregation, adhesion, and abnormalities captured in the images can be used as diagnostic indicators for blood stasis, and clearly advocates that endothelial cell function damage is indeed the essence of blood stasis [9]. This experiment mainly focuses on the interdependence and mutual influence between eNOs and endothelial cell functions. It is well established that under normal physiological conditions, NO act as an efficient vasodilator and its desired levels have a protective effect on endothelial cells. The activity of eNOs also influences the expression level of NO. Thus, eNOs plays a major role in the protection of endothelial cells [10]. The phosphatidylinositol-3 kinase-protein kinase B (PI3K-AKT) signaling pathway has a vital role in regulating cell proliferation, differentiation, and apoptosis. It also participated in protecting cells under stress and also affected the expression level of downstream eNOs [11–13]. Hence, in our study, the PI3K-AKT-eNOs signal pathway was selected for follow-up experiments.

*Chuanxiong (Ligusticum chuanxiong Hort.)* is a traditional Chinese herb medicine popularly used for activating blood and removing blood stasis. It possesses a variety of active compounds, such as phthalides, phenolic and organic acids, alkaloids, and polysaccharides. These components are commonly employed for promoting blood circulation and removal of blood stasis and related diseases [14, 15]. Levistilide A (LA) is one of the phthalides in the active ingredients of chuanxiong. It is mainly effective as antifibrosis, attenuates the expression of inflammation-related proteins, and also inhibits liver cancer cells [16–19]. However, studies on LA especially on blood stasis are unclear. Being an active ingredient of the chuanxiong, it may promote blood circulation and remove blood stasis and hence its role in blood stasis and endothelial cell protection needs to be explored.

This study intends to replicate the acute blood stasis animal model and establish a human umbilical vein endothelial cell (HUVEC) injury model to explore the effect of LA on acute blood stasis and its protective effect on endothelial cells and explore possible mechanisms involved to rectify blood stasis, to provide a reference to further researches on *Chuanxiong*.

## 2. Materials and Methods

### 2.1. Experimental Method

#### 2.1.1. Drugs and Reagents

LA was purchased from Chengdu Chroma Biotechnology Co., Ltd. BCA protein assay kit, RIPA buffer, and high sensitivity ECL chemiluminescence kit were purchased from New Cell & Molecular Biotech Co. Ltd. The IHC kit (KGSP01-KGSP02), eNOs (bsm-33176M), AKT (60203-2-lg), GAPDH (abs132004), PI3K (bs-10657R). Aspirin enteric-coated tablets (Bayer, Germany) and 0.1% hydrochloric acid epinephrine injection were purchased from the market.

#### 2.1.2. Animals

Male Sprague Dawley (SD) rats were purchased from the Laboratory Animal Center of Southwest Medical University and were raised at 22°C to 24°C room temperature, 40%–50% relative humidity, and a 12-hour light/dark cycle. The procedures were performed according to the guidelines provided by Experimental Animal Ethics Committee (Southwest Medical University, Luzhou, China). SD rats weighing 300g (*n* = 36) were selected for establishing the acute blood stasis model and *n* = 9 served as control. Animal experiments were initiated after 7 days of their adaption in a new environment.

#### 2.1.3. Experimental Design and Treatment

Forty-five rats were randomly divided into five groups (*n* = 9 per group) including the control, the model group, the model + aspirin group (Aspirin, 120 mg/kg), and the model + LA groups (LA, 5 mg/kg or 10 mg/kg). All rats received corresponding treatment intragastrically once a day for seven consecutive days. The control and the model groups were administered the same volume of normal saline. For the first 6 days, 40 minutes after the daily administration, except the control group, other rats were subjected to swimming exhaustion session until 50% of the rats settled naturally and stopped swimming. After 40 minutes of the seventh administration of the drug, all groups except the control group were given subcutaneous injection of 0.1% hydrochloric acid epinephrine (0.8 mg/kg). After two hours, they were again soaked into ice water (0–5°C) for 5 minutes, and after further two hours, the same dosage of adrenaline was injected.

All groups were fasted for 24 hours, received the last intragastric administration, and were anesthetized (i.p.) 40 minutes later with 10% chloral hydrate(0.3 mL/kg). Blood was collected with plastic vacuum tubes from aorta ventralis and used to determine pharmacodynamic indexes as described below (the doses of drugs and treatment duration were chosen based on the results of relevant published studies and our preliminary experiments).

#### 2.1.4. Detection of Pharmacodynamic Indices

The pharmacodynamic indices in blood were selected to evaluate the effects of LA on the acute blood stasis of rats. Coagulation indices were assessed by measuring the thrombin time (TT) and prothrombin time (PT), activated partial thromboplastin time (APTT), fibrinogen (FIB), and international normalized ratio (INR). The hemorheology indexes were measured by plasma viscosity and whole blood viscosity of low, medium, and high shear rates (1/s, 50/s, 200/s).

#### 2.1.5. Morphological Analysis and Immunohistochemistry of Carotid Arteries

*(1) Morphological Analysis*. Carotid arteries were dissected from all groups of rats, fixed in paraformaldehyde (4%), and embedded, and the sections prepared were analyzed for any morphological changes occurred in endothelium using hematoxylin and eosin (HE) staining.

*(2) Immunohistochemistry*. The sections (4 *μ*m) were obtained from paraformaldehyde (4%)-fixed, paraffin-embedded rat carotid arteries for IHC staining. The slices were treated with citrate antigen retrieval solution at 95°C for 15 minutes and washed three times with PBS. Next, the slices were treated with H_2_O_2_ (3%) solution for 10 minutes to inactivate the enzyme. Reagent-A (10% goat serum blocking solution) was added to the tissue section (completely covering) and incubated for 10 minutes. After draining, an appropriate amount of primary antibody (eNOs 1 : 500) was added to each slice and incubated overnight in a wet box at 4°C before washing 3 times with PBS. Reagent-B (anti-mouse biotinylated secondary antibody) was used to incubate the slices for 10 minutes and, thereafter, the incubated slices were again incubated with reagent-C (streptavidin-labeled HRP) for 10 minutes. Finally, freshly prepared DAB color-developer solution was added to each slice and the slices were kept at room temperature for 2–10 minutes before the slices were observed using an optical microscope.

#### 2.1.6. In vitro Studies

Human umbilical vein endothelial cells (HUVECs) were used as a model for cell experiments, and H_2_O_2_ was used for the induction of damage. The cells were divided into control, model, and LA-pretreated groups (5 *μ*mol/L). The CCK8 assay was used to detect cell viability and western blotting was used to verify the expression of related pathway proteins.

#### 2.1.7. Western Blot

After adding the serum-free F12K complete medium for 24-h synchronous treatment, the LA-containing drug culture medium was used for 24-h intervention. Then, the H_2_O_2_-containing culture medium was used for 4-h intervention. Cell lysate (120 *μ*L) was added and lysed on ice for 30 minutes after washing twice with PBS and centrifuged (12000 rpm) for 10 minutes at 4°C. The resulting supernatant was assessed for total cell proteins (the above operations were performed on ice). The protein concentrations were determined by the BCA method. The proteins were fractionated using polyacrylamide gel (10%) and were electrotransferred onto PVDF (0.45 *μ*m) membranes. After blocking with tris-buffered saline containing nonfat milk (5%), the membranes were incubated with primary antibody (diluted in accordance with the instructions) at 4°C overnight, and, thereafter, incubated with secondary antibodies, and finally band intensities were quantified by densitometry. The results were normalized using GAPDH (1 : 5000) as an internal standard.

### 2.2. Statistical Analysis

The SPSS17.0 software package was used to analyze data; the results were expressed using the mean ± standard error of mean (mean ± SEM). The data were analyzed using one-way analysis of variance, and the comparison between groups was operated by the least significant difference. *P* value of less than 0.01 or 0.05 was considered to be statistically significant.

## 3. Results

### 3.1. Performance of Rats after Modeling

**Figure 1:**
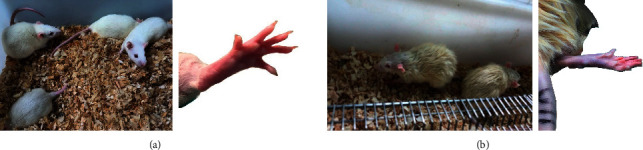
The appearance of rats after modeling. (a) The control group; (b) the model group.

Compared to the control group, the hair in the model group of rats appeared dim and dark yellow, and the claw nails appeared dark red and slightly purplish, showing clustering and chills (Figure 1).

### 3.2. Detection of Pharmacodynamic Indices

**Figure 2:**
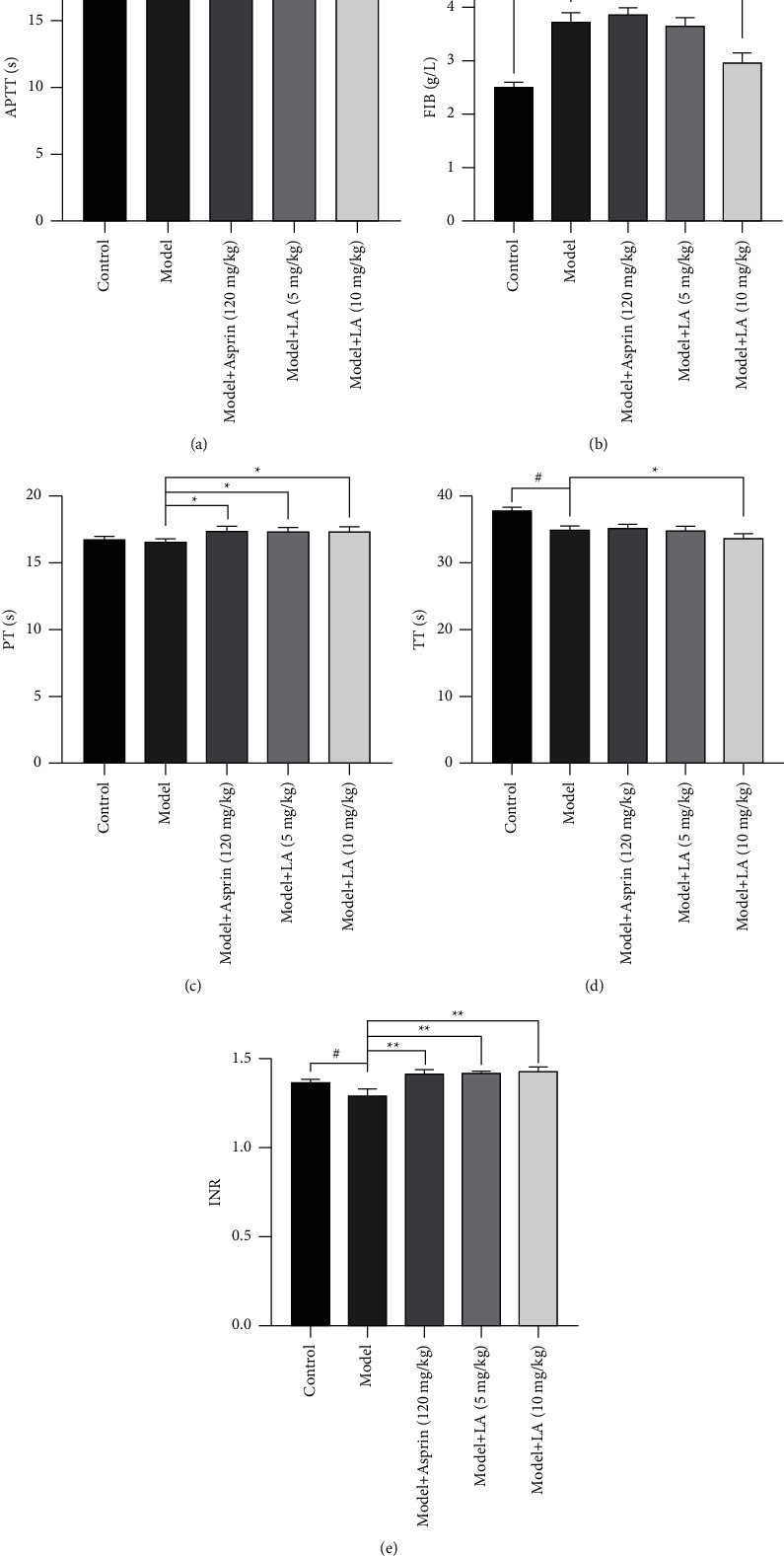
The effect of LA on coagulation parameters in the rat model with acute blood stasis. The values are mean ± SEM (*n* = 7–9) for (a) APTT (activated partial thromboplastin time), (b) FIB(fibrinogen), (c) PT (prothrombin time), (d) TT (thrombin time), (e) INR (international normalized ratio). The symbols in the charts represent the following: ^#^*P* < 0.05,^##^*P* < 0.01 as compared with the control group; ^*∗*^*P* < 0.05,^*∗∗*^*P* < 0.01 as compared with the model group.

Compared to the control group, the TT of the model group was shortened (*P* < 0.05), the FIB content increased (*P* < 0.01), and the INR decreased (*P* < 0.05). Compared to the model group, the PT and INR of the model + aspirin group increased (*P* < 0.01). The PT of rats in the model + LA(5 mg/kg) group was prolonged (*P* < 0.05), and the INR increased (*P* < 0.01). In the model + LA (10 mg/kg) group, PT prolonged (*P* < 0.01), APTT, TT shortened, FIB decreased (*P* < 0.01), and INR increased (*P* < 0.01). See Figure 2 for the determination results of plasma coagulation indexes of rats in each group.

**Figure 3:**
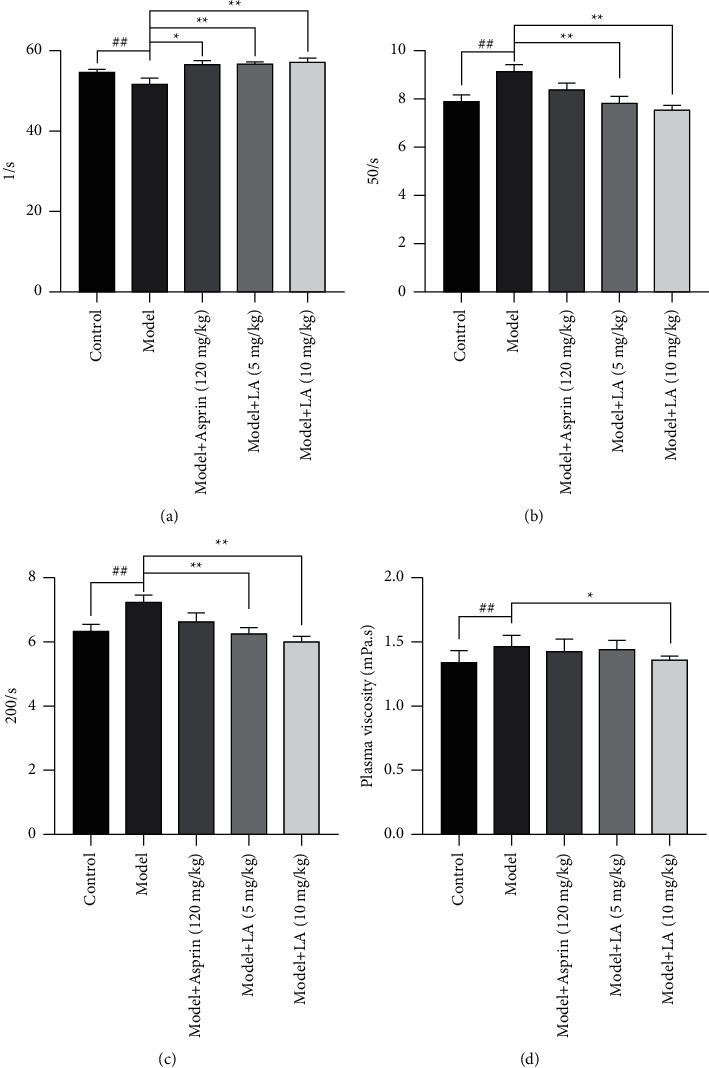
The effect of LA on hemorheological parameters in the rat model with acute blood stasis. The values are mean ± SEM (*n* = 6–7) for (a) 1/s, (b) 50/s, (c) 200/s, (d) plasma viscosity. The symbols in the charts represent the following: ^##^*P* < 0.01 as compared with the control group; ^*∗*^*P* < 0.05 and ^*∗∗*^*P* < 0.01 as compared with the model group.

Compared to the control group, the blood viscosity (*P* < 0.05) and the shear rate of whole blood viscosity (*P* < 0.01) of rats in the model group were increased. In comparison with the model group, the shear rate of the whole blood viscosity of rats in the model + aspirin group was decreased (*P* < 0.05). Likewise, in model + LA (5 mg/kg, 10 mg/kg)-treated groups, the shear rate and whole blood viscosity of rats were also decreased (*P* < 0.05 and *P* < 0.01). The hemorheology indices of rats are presented in Figure 3.

### 3.3. Carotid Artery Integrity

#### 3.3.1. Hemotoxylin Staining

**Figure 4:**
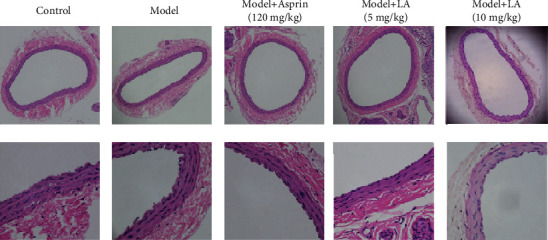
Hemotoxylin staining of rat carotid arteries. The upper and lower rows of pictures under the light microscope are 100× and 400×, respectively.

There is a phenomenon of shedding of carotid artery endothelial cells in the model group. On the contrary, the carotid artery endothelial cells in the treatment groups (aspirin and LA) were undamaged and smooth (Figure 4).

#### 3.3.2. IHC Staining

**Figure 5:**
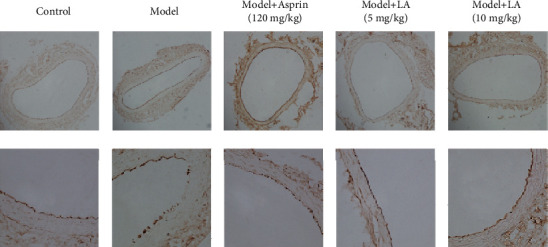
IHC staining of carotid arteries in each group of rats labeled with eNOs. The upper row is the view of 100×, and the lower row is the view of 400×.

The eNOs is expressed in endothelial cells and participates in the production of NO. Hence, in the carotid artery endothelium, it was labeled for IHC stain. In comparison with the control group (Figure 5), the positive expression of eNOs in carotid artery endothelial cells in the model group was discontinuous and defective, while in the treatment groups (aspirin and LA), they were relatively complete and continuous.

### 3.4. The Result of Cell Viability with CCK8 Assay

**Figure 6:**
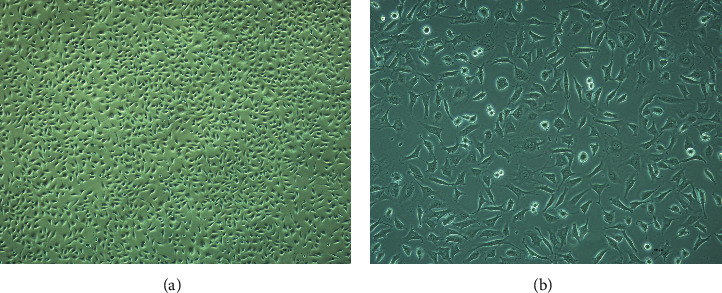
Morphology of HUVECs. (a) 40×; (b) 100×.

HUVECs represent the monolayer of cells with irregular morphology, which are round or oval, fusiform, or polygonal. After the fusion of cells into a sheet form, they appear as a “paving stone” arrangement (Figure 6).

**Figure 7:**
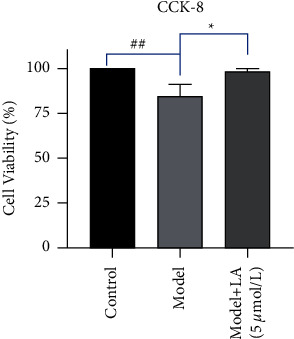
Effects of LA pretreatment on the cell vitality of HUVECs. The symbols in the charts represent the following: ^##^*P* < 0.01 as compared with the control group; ^*∗*^*P* < 0.05 as compared with the model group.

The cell viability of the model group decreased (*P* < 0.01) as compared to the control group, while in the LA-pretreated group, it is higher (*P* < 0.05) than that of the model group (Figure 7).

### 3.5. Western Blotting

**Figure 8:**
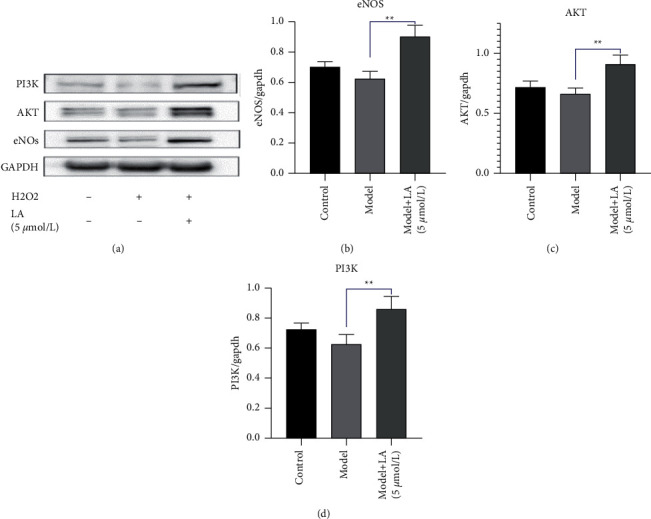
The protein expression level of eNOs, PI3K, and AKT in the control, the model, and the LA groups. The sample of each group was prepared three times, and the experiments were repeated at least three times. GAPDH was used as a loading control: (a) the expression level of each group, (b) eNOs/GAPDH, (c) Akt/GAPDH, (d) PI3K/GAPDH. The symbols represent: ^*∗∗*^*P* < 0.01 vs the model group.

After the extraction of proteins, their concentration was determined using the BCA method and the expression levels of PI3K, AKT, and eNOs of each group were detected by western blotting. The expression levels of PI3K, AKT, and eNOs in the LA-pretreated group were increased (*P* < 0.01) as compared to the model group (Figure 8).

## 4. Discussion

In modern medicine, blood stasis is related to microcirculation disorders, platelet aggregation, coagulation, and abnormal hemorheology [20, 21]. What's more, it has a close association with endothelial cell injury, which is also an initiating factor for various cardiovascular and cerebrovascular diseases. Therefore, it is of utmost significance to explore for other avenues to overcome endothelial-related damages, hence minimizing the induction of related disorders/diseases.

### 4.1. Establishment of Blood Stasis Rat Model

With the advancement of medicine, the combination of traditional Chinese and Western medicine to address the most appropriate treatment regimen has been increased and gaining popularity and confidence of researchers and the patients. In this context, the use of animal models for blood stasis has been improved substantially and the combination of TCM with modern medicine to replicate the combination of disease and syndrome modeling is challenging [22]. It is obvious that the successful replication of animal models is the cornerstone of subsequent research, and the efforts are underway for merging modern experimental methods with TCM to unravel its main indicators [23].

According to the philosophy of TCM, various animal models of blood stasis are classified according to their etiology and pathogenesis, such as *Qi* deficiency, Qi stagnation, blood deficiency, cold coagulation, phlegm turbidity, heat toxin, *yang* deficiency and *yin* deficiency, and aging [24, 25]. In our experiment, the method of “exhaustive swimming + adrenaline injection combined with ice water bath” was used to replicate the model. This strategy combined the theory of TCM, as swimming exhaustion may cause *Qi deficiency* in rats, and the administration of adrenaline stimulates the emotional changes of the human body when irritated or angry. It occurs because its action on adrenergic nerve receptors leads to the excitation of *α* and *β* receptors. As a consequence, the heart rate is accelerated but the demand of oxygen consumption is also increased in the myocardium, and it also induces the constriction of skin and mucosal blood vessels including blood capillary blood. Under the ice water bath conditions (simulating the invasion of “cold evil” according to the theory of TCM), the peripheral blood vessels will contract strongly, thereby increasing the afterload of the heart. The combined effect of several factors will result in a poor blood flow and increased blood viscosity leading to blood stasis [26, 27].

The model-building cycle in our experiments was 8 days. One week before model building, the rats in the model group were allowed to adapt to the water environment through swimming training. In the first 6 days of modeling, the rats were treated with exhaustion after gavage. Swimming was stopped when 50% of rats are unable to emerge to the surface of water on their own.

After intragastric administration on the 7th day, an ice water bath combined with epinephrine injection was given. On the 8th day, the samples were collected after intragastric administration. After model building, the rats were observed for clustering, chills, less movement, loose stools, and dark red claws, and the hair appeared dim.

The blood analysis showed that FIB was increased, whereas INR values were decreased, indicating that the blood is in a hypercoagulable state. The whole blood viscosity is another indicator of blood fluidity, and the increased plasma viscosity and various shear rates of whole blood viscosity suggest that the blood is in a highly viscous state [28]. Thus, with the combination of the above indicators and characterizations, it is convincing that the rat model of acute blood stasis has been successfully replicated.

### 4.2. In Vivo

A large number of studies have shown that the phytoconstituents of the traditional medicine are actively involved in the enhancement of blood circulation and removal of blood stasis and improvement in the aggregation ability of red blood cells and platelets, as well as reducing the red blood cell deformities. Furthermore, it may also play a crucial role in modifying blood coagulation and rheology indicators, for example, turmeric oil, mugwort oil, and tanshinone [29–33]. There are reports that many constituents probably are protecting the endothelial cells. These components not only play a role in biochemical indicators, but also have a certain protective effect on vascular endothelial cells, such as Z-bisaosterone and *β*-boswellic acid. [34–38]. It is known that LA possesses antifibrosis, antioxidation, and anti-inflammatory properties [39, 40]. However, its efficacy in the blood stasis is still unclear, and we have made an attempt to explore it. The rat whole blood from all the groups was collected, and the blood coagulation indicators (PT, TT, APTT, FIB, INR) and the plasma viscosity, as well as shear rates of whole blood viscosity (1/s, 50/s, 200/s), were evaluated.

Our results demonstrated that after treatment with LA, PT was prolonged, whereas FIB levels were decreased and INR values were increased. What is more noticeable is that the plasma and whole blood viscosity after LA treatment were reduced in low, medium, and high shear rates (1/s, 50/s, 200/s). Thus, the intervention of LA has more likely improved the hypercoagulable and hyperviscous state of blood by interaction with the coagulation pathway, thereby improving the hypercoagulable state of blood.

The maintenance of the normal structure and function of the vascular endothelium is of great significance to the homeostasis of the internal environment. The disruption of balance may lead to endothelial dysfunction and endothelial cell shedding, which is a major contributing factor for poor blood circulation, microcirculation disorders, and even thrombosis. Based on the aforementioned reasoning, the carotid arteries from rats were analyzed for morphological examinations. The HE staining and eNOs-labeled IHC staining revealed that in the LA treatment group, carotid endothelial cells were arranged regularly, smoothly, and continuously. Moreover, the expression of eNOs was elevated than the diseased model group. Taking together, our finding demonstrates that LA intervention has certainly played a protective role regarding the integrity of endothelial cells.

### 4.3. In Vitro

The HUVECs (often selected as models for the study of cardiovascular and cerebrovascular diseases) were selected for *in vitro* experimental verification. The application of TCM in blood stasis and injured endothelium has gradually increased. The protective effect of TCM and its components on endothelial cells is well recognized [41–43]. Most studies demonstrated the involvement of the PI3K-AKT signaling pathway, among which the expression of eNOs is regulated affecting the NO levels [44–46]. This signaling molecule acts widely in the body involved vasodilation and circulation, regulation of inflammation, and platelet activity, while ET has a strong vasoconstrictor effect. Under physiological conditions, a dynamic balance between these two molecules is maintained [47, 48]. Under injurious endothelial cells, their secretory function becomes impaired leading to reduction in NO and increment in ET-1 (the most important subtype in the cardiovascular system) levels with increased endothelial permeability, causing poor blood circulation and platelet adhesion, eventually leading to pathological conditions such as microcirculation disorders and thrombosis [49].

In the present study, the CCK8 experiments were carried out to screen the appropriate concentration of LA on HUVEC cells. After the pretreatment of cells with LA cells, H_2_O_2_ was added as an intervention factor. The cell viability of the LA-pretreated group was higher (*P* < 0.05) than that of the model group, implying that the intervention of LA has an ability to increase the activity of endothelial cells. To further the investigation, the expression levels of PI3K, AKT, and eNOs were determined. The expression levels of these proteins in the LA-pretreated group were increased, supporting that LA is probably involved in the regulation of the PI3K-AKT pathway, thereby affecting the expression level of eNOs and controlling the levels of NO, which certainly has a protective effect on endothelial cells.

### 4.4. Summary and Outlook

Vascular endothelial cells have an ability to directly sense the pressure of the blood vessel wall and release a variety of biologically active molecules to maintain physiological stability. If dysfunction occurs, it may cause abnormalities in blood pressure, blood lipids, and secretory functions, leading to the occurrence and development of various pathological conditions [50, 51]. Hemorheology is a comprehensive indicator of blood flow characteristics. The related indicators such as blood viscosity and coagulation provide a theoretical basis for the diagnosis and treatment of diseases [52, 53].

It is well established that blood stasis leads to abnormal hemorheology, accompanied by endothelial cell damages. We have verified the relationship between blood stasis and hemorheology and endothelial cell injury through *in vivo* and *in vitro* experiments, and then verified that LA can improve the abnormality of the indicators above to a certain extent and also primarily explored the mechanism of protection via the PI3K-AKT signaling pathway. However, in order to further study the efficacy and related mechanisms of LA, it is planned to investigate the downstream products and related molecules of eNOs. At the same time, the mechanism (s) for improving blood viscosity and coagulation needs to be explored in the animals.

## 5. Conclusions

It is concluded that LA is an important component of chuanxiong. In the SD rat model of acute blood stasis, it has increased the blood viscosity caused by blood stasis, improved the hypercoagulable state of blood, through morphology, and protected vascular endothelial cells. It was also verified that LA can increase the activity of endothelial cells and has a protective effect on injured endothelial cells through the PI3K-AKT-eNOs signaling pathway.

## Data Availability

All the data generated or analyzed during this study are included in this published article.
